# Can innovation strengthen resilient, just and sustainable health systems in disaster-prone settings? Insights from HSR2024

**DOI:** 10.1093/heapol/czag036

**Published:** 2026-06-29

**Authors:** Nanuka Jalaghonia, Rumana Huque, David Daniels

**Affiliations:** Partnerships & Engagement Manager at Health Systems Global, MannionDaniels, Universal House, 1-2 Queens Parade Place, Bath BA1 2NN, United Kingdom; Professor in the Department of Economics, University of Dhaka, Bangladesh; Executive Director at ARK Foundation, Bangladesh / Suite C-3 & C-4, House 06 Rd 109, Dhaka 1212, Bangladesh; Managing Director, MannionDaniels, Universal House, 1-2 Queens Parade Place, Bath, BA1 2NN, United Kingdom

**Keywords:** health systems, health system resilience, innovation, digital health, artificial intelligence, community engagement, climate change, disaster preparedness, fragile and conflict-affected settings, participatory design, health information systems, governance, equity

## Abstract

Health systems in disaster-prone settings face recurrent shocks that expose and often deepen existing inequities. Increasingly, innovation, particularly digital technologies and artificial intelligence (AI), is positioned as a pathway to strengthen resilience, responsiveness, and accountability. Drawing on insights from innovation-focused sessions at the 8th Global Symposium on Health Systems Research, this commentary examines whether and under what conditions innovation can contribute to resilient, just and sustainable health systems. We define disaster-prone settings as contexts repeatedly exposed to acute shocks and chronic stressors, such as climate events, outbreaks, and displacement, where service delivery is periodically disrupted and recovery shapes long-term system trajectories. Across diverse examples, including digital dashboards, interoperable data systems, AI-supported decision tools, and community-driven innovations, the symposium highlighted how innovations can improve detection, coordination, and service continuity, particularly during crisis conditions. These approaches can make populations previously invisible to the health system visible, strengthen real-time decision-making, and support anticipatory action. However, the analysis shows that innovation does not inherently produce equitable outcomes. Digital and AI-enabled tools may reproduce or even intensify existing exclusions if they rely on unrepresentative data, lack interoperability, or operate without transparent governance and accountability. Many technologies remain at an early stage, with evolving evidence on effectiveness and equity impacts, placing policymakers in a position of making decisions in uncertainty. In disaster contexts, where rapid decisions and weakened oversight are common, these risks are amplified. We argue that innovation strengthens resilience and justice primarily when accompanied by institutional readiness and governance capacity. This includes clear mandates, regulatory frameworks, ethical safeguards, and mechanisms for iterative learning that translate evidence into practice. Equally important are participatory approaches that ensure communities shape design and decision-making, rather than being passive data sources.

Key messages:

- Digital and artificial intelligence (AI)-supported innovations have the potential to contribute to faster detection, coordination, and accountability during crises, but their equity effects depend on context-sensitive design, representative data, interoperability, and transparent oversight. Without these, they may reproduce exclusions or narrow equity perspectives even when system performance improves.
- Many technology tools are still at an early stage, often with recognized shortcomings, highlighting the need for regulatory frameworks, ethical safeguards, and accountability mechanisms that prevent new technologies from reinforcing existing inequities. Policymakers should prioritize investments in research, innovation, and capacity building to support safe and equitable adoption.
- Policymakers must remain attentive to trade-offs, uncertainties, and unintended consequences, adopting flexible regulatory and implementation approaches that allow iterative learning as digital and AI tools and their capabilities continue to develop. This requires institutional readiness, clear mandates, financing rules, and decision pathways so that learning translates into changed practice rather than repeated pilots.

## Introduction

The 8th Global Symposium on Health Systems Research (HSR2024), held in Nagasaki, Japan, featured innovation-focused sessions examining how health systems research is responding to growing inequities, environmental shocks, and governance challenges in disaster-prone settings. Here, ‘disaster-prone settings’ refers to contexts repeatedly exposed to acute shocks and chronic stressors, such as droughts, floods, outbreaks, conflict-related displacement, and infrastructure disruption, where routine service delivery is periodically overwhelmed and where recovery and adaptation shape long-term system trajectories. These discussions emphasized practical approaches that strengthen resilience and justice by improving information access and flows, widening participation in system design, and supporting inclusive, trusted, and community-responsive decision-making that can hold under shock conditions, not only in stable periods.

Drawing on these sessions, this commentary contributes to the supplement ‘Changing Health Systems: Advancing Justice and Sustainability through Research, Policy and Practice’ and speaks directly to its core concerns by examining innovation not simply as technological advancement but as a pathway through which justice and sustainability are pursued and, at times contested amid rapid system change. It argues that while digital tools, artificial intelligence (AI)-enabled governance, and data-driven planning can improve coordination and responsiveness, they can also oversimplify complex realities, narrow equity perspectives, or reproduce existing exclusions if not carefully contextualized. In disaster-prone settings, innovation strengthens resilience and justice especially when governance, accountability, and decision-making authority are deliberately reshaped under shock conditions to support inclusive, timely, and trusted action. The selected examples below illustrate what this looks like on the ground and what it implies for long-term system functioning.

In many different contexts, innovations in digitalization and data use demonstrated how long-standing information gaps are being addressed by making marginalized and hard-to-reach populations more visible to the system. Research in Nairobi's Mathare informal settlement provides an example where a community-designed physical address system combined participatory mapping, locally defined address codes, and wayfinding markers to improve access, navigation, and referral where formal addressing is absent. In disaster settings, when facilities are disrupted, populations move, or rapid outreach is needed, such locally anchored ‘addressability’ can become a practical resilience asset for targeting services, referrals, and follow-up. In parallel, digital health dashboards showed how real-time, actionable data can support day-to-day system management: one dashboard, built through a progressive web application, enabled anonymized citizen reporting to help authorities track emerging health trends, manage risks, and respond more quickly while also creating a two-way channel between communities and decision-makers (Katapally and Ibrahim 2023, Kori et al. 2025). Making previously invisible populations visible in information systems can support more equitable service delivery, but whether new data translate into fairer allocation depends on resources, mandates, and political will. As a result, populations may be newly ‘seen’ in data yet remain underserved in practice, reinforcing existing hierarchies in access and response. In early implementation, uneven skills and confidence in using new digital tools can also shape how evidence is interpreted and acted upon and may limit decision-makers’ ability to assess the quality, bias, and relevance of underlying datasets.

Several symposium sessions moved beyond ‘point solutions’ to explore interoperability and system-wide integration, highlighting what it takes for digital investments to improve continuity of care and routine decision-making and to maintain continuity when shocks disrupt normal pathways. Indonesia's SATUSEHAT platform illustrated that it is feasible to integrate electronic medical records and health data from hospitals, clinics, laboratories, and pharmacies into a national repository; thereby reducing fragmentation and repeated data entry while supporting referrals through access to diagnostic results, procedures, and medication history across facilities (Azhar 2023). Somalia's work to include private sector data in the national Health Management Information System allowed a different route to similar goals, focusing on data-sharing protocols, interoperability standards, and approaches to engaging private providers to improve surveillance, utilization tracking, and health financing insight. Across these examples, the shift was from retrospective reporting toward real-time reporting and more forward-looking system steering. It is proposed that such integration can strengthen procedural justice by improving continuity, accountability, and transparency across fragmented service landscapes. In disaster-prone settings, equity implications are often more pronounced: fragmented records and weak interoperability disproportionately penalize those who move, lose documentation, or depend on episodic emergency care.

Within this broader digital agenda, AI emerged as a distinct strand. Sessions suggested AI is being used in at least three ways: strengthening governance and accountability, supporting frontline decision-making, and linking climate risks to health planning. To keep the disaster lens central, these uses are most consequential when they shorten the time between signal and action during shocks, detecting emerging risks, triaging scarce capacity, and supporting targeted protection of those most exposed. In Uttar Pradesh, India, the eKavach initiative illustrated how AI-enabled alerts and automated monitoring can flag potential service delivery or performance issues for follow-up by managers (National Health Mission 2022). In peri-urban Sindh, Pakistan, telehealth clinics integrating an AI-supported digital decision support system described how providers were trained to use decision tools during consultations, aiming to improve diagnostic support and perceived quality of care in low-resource telehealth settings (Tahir et al. 2024).

Importantly, sessions did not treat AI as inherently beneficial; they repeatedly underlined that AI contributes to resilience only when it is governed and embedded responsibly. Discussions on responsible AI and stewarding sustainable digital transformation stressed data security, privacy safeguards, bias mitigation, and transparency about how tools shape decisions, clear accountability, and the importance of maintaining human oversight. Country experiences, such as digital health reforms and regulation discussed from Georgia and Kuwait, reinforced a practical point: trust, legitimacy, and institutional alignment often determine whether AI-enabled tools become durable parts of a health system or remain short-lived pilots (Gotsadze et al. 2024, Alibrahim et al. 2025). These governance considerations highlight that AI can either strengthen or undermine justice depending on how authority, accountability, and oversight are structured. Where data representation is uneven or oversight is weak, algorithmic systems risk reproducing the very inequities they seek to address in service prioritization, surveillance, and resource allocation. In disaster-prone settings, these risks intensify because emergency decisions are faster, oversight can be weakened, and ‘exceptional’ measures can become normalized, making accountability architecture (not only tool performance) the core resilience question.

Equally prominent, often alongside digital innovation, were innovations grounded in community engagement, traditional knowledge, and participatory design. Across sessions, presenters emphasized that technical solutions alone are rarely sufficient to build uptake and trust, particularly in marginalized or fragile settings. Examples illustrated how community narratives, indigenous languages, and co-designed service pathways can counter misinformation, improve acceptability, and strengthen relationships between communities and providers. In Puno, Peru, co-developed messaging in indigenous languages and trusted local channels were used to strengthen engagement during COVID-19 (Samuel et al. 2026). In Isiolo and Marsabit counties in Kenya, participatory storytelling drew on Indigenous narratives to support maternal and child nutrition practices and build trust between communities and providers (Kinyili et al. 2024). These approaches can strengthen legitimacy by recognizing lived experience and local knowledge, but participation does not automatically shift power. If marginalized groups are not meaningfully included, or if local knowledge is gathered without influence over decisions, the result can be consultation without change, while biomedical and managerial priorities continue to dominate. Across these sessions, innovation was less about introducing entirely new interventions and more about strengthening how systems listen, adapt, and learn by widening participation in design, validating multiple forms of knowledge, and improving the fit between services and the communities they aim to serve. Such participatory processes can support accountability and shared decision-making but only if communities have sustained influence rather than being engaged through time-limited project cycles.

A cross-cutting theme across the discussions was the shift from single datasets or single interventions towards joined-up intelligence, connecting health information with environmental and social signals, to support preparedness and adaptive response. In this strand, innovation was about creating practical linkages between data, institutions, and decision points so that early signals could translate into timely action.

In South Africa, the session on the health sector's response to the Cape Town drought highlighted how real-time documentation of measures, such as securing water through boreholes and promoting hygiene behaviour change, can generate operational learning during crisis periods, while also showing the challenge of institutionalizing those lessons into routine policy once the shock has passed (Quintana et al. 2025). In Nepal, sessions on sub-national climate resilience described how provincial and district systems are integrating climate considerations into health planning through climate-smart infrastructure, budgeting and decentralized early warning, often requiring coordination between health authorities, meteorological services and community health workers to make information actionable at service-delivery level (Dhimal et al. 2025). In the Philippines, the dengue modelling showed how linking climate and epidemiological data can estimate temperature-mediated dengue burden and support more proactive decisions, including targeting vector control and allocating resources to higher-risk areas (Seposo et al. 2024). By enabling anticipatory action, these approaches help reduce vulnerability and support more equitable protection of at-risk populations, provided early warning and response capacities are equitably distributed.

Taken together, the sessions not only demonstrated important operational potential in AI and digital innovation but also made clear that technological change is moving faster than the institutional arrangements meant to govern it. Much of today's AI in health remains early stage and will evolve quickly, which complicates policy decisions about whether to adopt, how to regulate, and what to scale in crisis contexts. Policymakers are asked to judge value and risk while evidence is still forming, and outputs may sometimes be misleading or not fully reliable, and while design choices and deployment conditions can change effectiveness and equity outcomes under shock. These choices also carry practical resource and productivity implications, including where staff time shifts and what new skills and support are required to use tools safely and well during surges. The forward-looking priority is not only more innovation, but also governance and learning arrangements that can keep pace, test assumptions, make trade-offs explicit, and strengthen accountability as technologies and use cases evolve in disaster-prone settings.

Alongside this, future progress will depend on whether data systems and algorithms are genuinely representative and context-sensitive, rather than capturing only partial realities. Strengthening collaboration between communities, researchers and policymakers will be essential to ensure that emerging digital and AI tools enhance equity, contextual understanding and inclusive decision-making rather than narrowing them.

This commentary does not attempt to cover every innovation presented at HSR2024. Instead, it synthesizes selected insights to show how innovation is being operationalized to strengthen health system resilience and justice, capacities widely recognized as essential for timely response, ongoing adaptation and fair protection, yet often discussed more than implemented (Barasa et al. 2017, Ghebreyesus et al. 2022). Across digital, AI-supported, community-led and climate-responsive initiatives, resilience and equity emerged less as fixed attributes and more as capacities built through learning, integration, and sustained system change. Their value depends on governance, whether systems are inclusive and accountable and whether participation, resources, and decision-making authority are distributed fairly. Taken together, these experiences suggest a shift away from technology-first or top-down models toward practice-driven innovation grounded in local realities and careful use-case design as tools and evidence continue to evolve. The evidence presented suggests that innovation strengthens resilient and just health systems only to the extent that it is matched by institutional readiness and governance capacity: the ability to scrutinize rapidly evolving tools, regulate uncertainty, maintain accountability, and translate new forms of data and analysis into action. It also depends on whether technological change redistributes, rather than recentralizes, authority, and operational capacity toward the levels that carry the burden of shock most directly, including frontline teams district managers and communities.

Under accelerated technological change, the future contribution of innovation to resilient, just and sustainable health systems will depend not primarily on the novelty of the tools themselves but on whether institutions are prepared to govern them credibly, monitor, and manage them over time, and prevent new forms of data, technical expertise and platform dependency from narrowing public accountability or displacing decision-making authority from those closest to the burdens of crisis.

The forthcoming 9th Global Symposium on Health Systems Research to be held in the Eastern Mediterranean Region (9–11 November 2026) will offer a useful opportunity to continue the debate on how digital transformation and AI are being introduced and governed across diverse and shock-prone settings.

## Reflexivity statement

Our authorship team reflects diverse perspectives across gender, seniority, and geography. Two authors hold senior leadership roles, and one is mid-career, with experience spanning research, programme management, and policy engagement. Collectively, the authors bring practical expertise in health systems strengthening across Georgia, Bangladesh, Nepal, Kenya, Nigeria, Somalia, and the UK, including work in public health and humanitarian settings, health financing and budgeting, tobacco control policy and advocacy, and capacity strengthening to support the conduct and use of health policy and systems research, alongside collaboration with governments, non-governmental organizations, international partners, and academic institutions.
